# A systematic review protocol for quantifying bycatch of critically endangered leatherback sea turtles within the Pacific Ocean basin

**DOI:** 10.1186/s13750-024-00352-3

**Published:** 2024-11-29

**Authors:** Anna A. Ortega, Nicola J. Mitchell, Nina Marn, George L. Shillinger

**Affiliations:** 1 The University of Western Australia, 35 Stirling Highway, Crawley, WA 6009 Australia; 2 Oceans Institute, The University of Western Australia, 54, Fairway, Crawley, WA 6009 Australia; 3 Upwell Turtles, 99 Pacific Street Suite 375-E, Monterey, CA 93940 USA; 4 Ruđer Bošković Institute, Bijenička Cesta 54, Zagreb, 10000 Croatia

**Keywords:** Sea turtle, Fisheries bycatch, Endangered species, Conservation planning

## Abstract

**Background:**

The Pacific Ocean supports two leatherback sea turtle populations, each of which is Critically Endangered primarily as a result of ongoing incidental bycatch within small-scale and industrial fisheries. Conservation planning has included population viability analysis (PVA), which depends on accurate data on mortality and morbidity (sublethal effects) rates to yield realistic results that can inform management decision-making. Existing leatherback PVAs are based on best available data, however, estimates of mortality and morbidity rates are heavily influenced by estimates of bycatch. These, in turn, are based on unknown levels of observer coverage in many fisheries, estimated to be less than 1% coverage in some artisanal and industrial fleets. Leatherback population recovery depends on bycatch reduction. It is vital to understand the source, scope, and scale of leatherback bycatch wherever and whenever leatherbacks occur. Here, we outline a protocol for a systematic review to aggregate existing estimates of leatherback bycatch within the Pacific Ocean, on a population- and basin-level. These results will generate the first comprehensive estimate of leatherback turtle bycatch for any ocean basin and will be incorporated into future conservation planning for Pacific Ocean populations.

**Methods:**

A Boolean search string will be input into several bibliographic databases to yield articles and grey literature (governmental, business, and industry information not controlled by commercial publishing) related to the research question. Additional grey literature searches, snowball sampling and expert elicitation will be used to create as robust and comprehensive a pool of literature and/or databases as possible. Retrieved articles will be reviewed for eligibility using the SPIDER search strategy tool (Sample— Phenomenon of Interest—Design—Evaluation —Research type; 7). Articles which meet the criteria will be included in the systematic review, and their data will be collated into comprehensive estimates of leatherback sea turtle bycatch within the Pacific Ocean, one for each population. These data will be further teased apart by fishery size, fishing gear type, fishing nation, fishery region, and fishery target species, to target management more directly. This information will be published and provided directly to stakeholders for use in conservation management.

**Supplementary Information:**

The online version contains supplementary material available at 10.1186/s13750-024-00352-3.

## Background

The rapid decline of nesting females in both the East and West Pacific populations of leatherback sea turtles (*Dermochelys coriacea*) has led to their listing as Critically Endangered by the International Union for the Conservation of Nature [35, 36]. Population declines in the past have been driven by nest disturbance and human harvest of eggs and adult turtles [35, 38], however conservation efforts to mitigate nesting beach threats have been extensive. The most prevalent current threat for Pacific leatherbacks is bycatch from commercial and small-scale fisheries, which is both unquantified and hard to manage [19].

Leatherback populations are susceptible to incidental catch by fishing vessels in the Pacific Ocean due to their highly migratory nature, and because of their long generation time, threats are detrimental to population persistence [37]. This bycatch can result in morbidity, which causes (sublethal) injuries, or mortality, which directly removes individuals from the population [16]. Leatherbacks are incidentally caught in a variety of fishing gear types (gillnet, longline, trawl), target fisheries (tuna, billfish), and fisheries scale (small-scale, to industrial) [33, 39]. For the purpose of this analysis, fishery categorisations follow the Intergovernmental Science-Policy Platform on Biodiversity and Ecosystem Services (IPBES; 17). A small-scale fishery refers to one that generally presents the following characteristics: low capital investment, family or community based high-labour activities, no/small sized vessels, relatively low production consumed locally or sold directly, and/or operating close to the shore on a single-day basis. In contrast, industrial fisheries display high capital equipment and expenditure, high levels of mechanisation, motorisation and onboard processing, large vessel size, and often have access to the global market, with offshore, multi-day operations [17]. Management of fisheries bycatch for migratory species is difficult because decisions must be consensual and consistently enforced between nations, regional fisheries management organisations, and conservation and convention entities [34]. As a result, leatherbacks continue to be removed from Pacific populations, and the rate at which this is happening remains unknown due to the lack of comprehensive data and the complexity of aggregating published bycatch data from observers and logbooks [33].

Despite the significant gaps in bycatch data for the Pacific Ocean, the mortality associated with bycatch has been estimated regionally for use in population viability analysis (PVA) within the East Pacific leatherback population [27]. PVA requires demographic and life history data that are specific to a population and uses these data to project future population trends and extinction risk, often based on alternative management strategies [25]. Use of PVA in conservation decision making has been limited due to data deficiency, prompting concerns that results may be spurious when estimated parameters are used as inputs [3, 8]. However, when a population is well studied, resulting in sufficient data to ensure that input parameters are accurate and population-specific, and if future change is predictable, then the predictive power of PVA is immensely valuable [32]. Extinction of the East Pacific leatherback population has been predicted by 2080 based on PVA (6, 24, 27), prompting urgent and diverse conservation efforts from a range of stakeholders [6]. However, the utility of the East Pacific PVA can be improved by quantifying leatherback bycatch within the Pacific Ocean. Similarly, for the West Pacific population of leatherbacks, a PVA has been crucial in setting bycatch limits to help manage fisheries in the United States longline fleet [23], and individual nesting beach trends have been analysed [26]. However, this work could be expanded to include all West Pacific subpopulations within one PVA model. In essence, all future PVAs for both East and West Pacific leatherbacks require comprehensive, basin-wide, and accurate estimates of fisheries bycatch, so that population trajectories can be revised to inform more targeted conservation actions.

Here, we propose a protocol for a systematic review of academic and grey literature to collate existing data in order to quantify the level of leatherback bycatch in the Pacific Ocean. The scope of previous literature reviews of fisheries bycatch can be categorised as follows:

i. Focal species: either several taxa (marine mammals, sea birds, sea turtles [4, 18, 22], one taxa (sea turtles (1,11,28,39,40, or one species (leatherbacks [10, 23, 33];
ii. Scale: either one fishery [4, 18, 23], one country [10, 11], one region [1, 28, 31], one ocean [33], or global [22, 39, 40];
iii. Gear type: either one type (e.g. longline [10, 23]), multiple types (e.g. net, longline, trawl; [22, 39, 40], multiple types with subcategories (e.g. longline (deep and shallow set) [23]), or all types [11];
iv. Data sources: either directly from fishers (logbooks; surveys [1, 28]), observers (18,31, or literature (22,39,40;
v. Goal: either to aggregate past bycatch [11, 39], create an extrapolated estimate of bycatch [1, 4, 28], or map the risk of bycatch [10, 33].

No two studies pursued identical methods; each used methods that were applicable to their goal and accepted the associated limitations. For example, including multiple gear types required standardisation of bycatch rates among gear types, often with a measure of bycatch per unit effort (BPUE). Risk maps have been favoured to tease apart the many predictor variables for bycatch, such as sea surface temperature, depth, and higher interactions probabilities along leatherback migration paths or in foraging/breeding grounds at certain times in a year [33]. As a result, the complexity of combining all available data has resulted in few published estimates of bycatch on global scales, but such an understanding is crucial to accurately assessing the threats to declining populations.

Our methods are similar to those used in a previous review of marine turtle bycatch [39], which included all seven marine turtle species, but only included three classifications of gear types, and excluded some data sources (logbooks). These constraints are similar to many bycatch reviews, which often search peer-reviewed literature for interactions between one gear type and numerous bycatch species [5, 20]. Here, we adjust these methods to focus on the impact of all gear types for one species, in one ocean-basin. However, regardless of the redefined scope, knowledge gaps about the level of bycatch in some fisheries remain. To address this, we’ve applied the methods used in fisheries catch reconstruction [30], which have not been previously applied for single-species bycatch analyses. Catch reconstruction methods aim to find (a minimum of) one source for each characteristic of interest by using all available data, while being transparent about the original source and reporting the confidence of each estimate [30]. This allows a comprehensive bycatch estimate that uses all available data, while accepting that there will be uncertainty in that estimate.

## Objective of the review

We will present all known information and estimates of leatherback bycatch in Pacific Ocean fisheries by aggregating information in an accessible format. This should serve as the basis for estimating the leatherback bycatch within the Pacific Ocean, and for further informing conservation management. To achieve this, the review will involve (1) categorization of bycatch by different fisheries (gear, nation, region, target species); (2) grouping of quantification by publication method (academic or grey literature); and (3) through meta-analysis, creating an understanding of discrepancies in data, highlighting knowledge gaps for future monitoring and/or research.

**Fig. 1:**
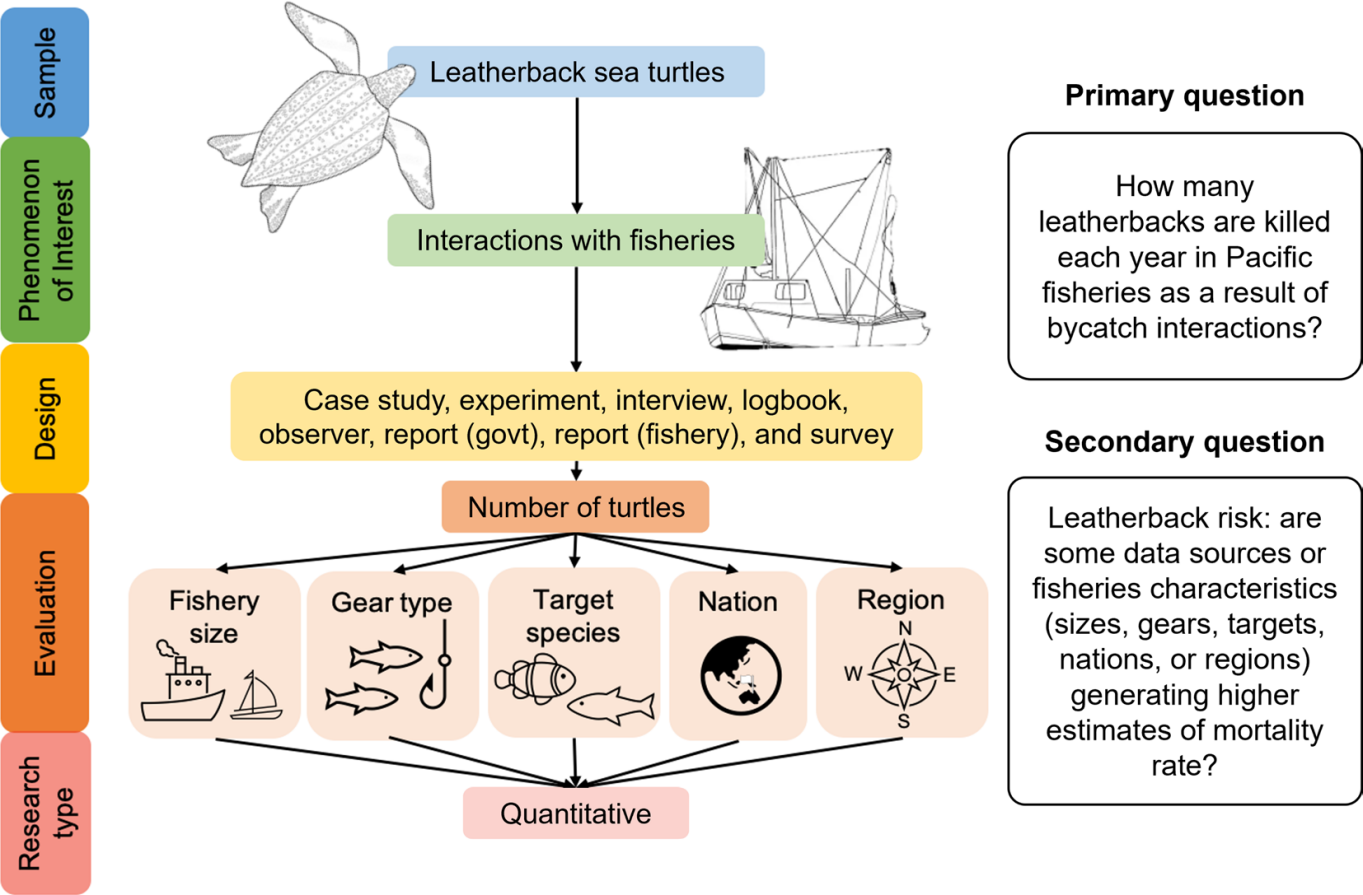
Visual representation of the SPIDER search strategy, as applied to this study with primary and secondary questions

This review utilises the SPIDER search strategy tool (Sample — Phenomenon of Interest — Design — Evaluation — Research type) to narrow the search to relevant literature [7] (Table 1). The primary question to be addressed through this systematic review is: *How many leatherbacks are killed each year in Pacific fisheries as a result of bycatch interactions?* And secondarily, to identify risks to leatherbacks: are some data sources or fishery characteristics generating high estimates of mortality rate? (Fig. 1)

**Table 1:** Study eligibility criteria based on the SPIDER search strategy tool framework

*Sample*	leatherback sea turtles; Dermochelys coriacea
*Phenomenon of Interest*	Individuals acting as a bycatch species; which are unintentionally caught during fishing activity or whose catch can be attributed to fisheries interactions (passive catch: abandoned nets) a. Morbidity (sublethal effects) versus mortality i. Mortality: retrieved dead from the net/line. ii. Morbidity: retrieved alive, potentially but hooked in the mouth or on the flipper. These will be further split based on gear type and expected post-release mortality. 1. Findings from this review will be utilised to create a better understanding of post-release mortality in different gear types, which will be applied to calculate post-release mortality.
*Design*	The number of leatherbacks caught by a fishery could be captured in many designs, as some data come from onboard observation (observers), and others from experiments to trial hook effects on bycatch (experiment). Included here are case study, experiment, interview, logbook, observer, report (govt), report (fishery), and survey designs.
*Evaluation*	Quantitative element: number of turtles per *characteristic* over time a. Characteristics included, to allow comparison i. Fishery size 1. Industrial, small-scale (40). ii. Fishing gear type 1. Direct take*, gillnet, longline, purse seine, squid jig/troll, trawl; identified as likely to be interacting with leatherbacks (2). * Refers to fisheries aimed at leatherbacks (Kei Islands, Indonesia). If specific in literature, opportunistic take of leatherback bycatch in any fishery will contribute to (1) both the bycatch estimate for that fishery, and (2) the “direct take” mortality estimate. iii. Fishery target species 1. Billfish, tuna; target species which interact with leatherbacks (33). * There are many target species which are not listed here, particularly within small-scale fisheries. Therefore, the Boolean search string does not require specific target species, but rather the term “bycatch”. All resulting target species will be recorded as metadata. iv. Nation (EEZs or international water fishers) 1. American Samoa, Australia, *Cambodia*, *Canada*, Chile, China, Colombia, Cook Islands, Costa Rica, *Dominica*, Easter Island, *Ecuador*, El Salvador, Fiji, French Polynesia, Galapagos Islands, Guam, Guatemala, India, Indonesia, Japan, Kiribati, *Malaysia*, *Marshall Islands*, Mexico, Micronesia, *Nauru*, New Caledonia, New Zealand, Nicaragua, Niue, Norfolk Island, *North Kore*a, Northern Mariana Islands, Norway, Palau, Panama, Papua New Guinea, Peru, Philippines, Pitcairn Island, Russia, *Samoa*,* Sinapore*, Solomon Islands, *South Korea*, *Spain*,* Taiwan*, *Thailand*, Timor Leste, Tokelau, Tonga, Tuvalu, *Ukraine*,* United Kingdom*,* Uruguay*, United States, US Minor Outlying Islands (Midway Atoll, Wake Island, Johnston Atoll, Kingman Reef, Palmyra Atoll, Howland Island, Baker Island, Jarvis Island), Vanuatu, *Venezuela*, Vietnam, Wallis and Fatuna, *Yemen* *Normal text denotes nations with Pacific EEZs; *italics* denote nations which do not have Pacific EEZs but have fishing vessels in the Pacific Ocean according to The Sea Around Us (29). v. Region 1. Pacific: North, Northeast, Northwest, South, Southeast, Southwest, East, West, Central
*Research type*	Quantitative; as qualitative methods are incomparable across a broad range of study methods and locations (34). a. Qualitative data will be noted when relevant, but its collection is not a primary objective of this work.

## Methods

### Scoping

To ensure that the chosen search strategy covers the desired scope (our characteristics of interest), an initial scoping exercise using a list of ten reference articles was performed. These articles were chosen in a preliminary literature review based on their combined ability to cover the characteristics of interest in this study.

- Fishery size (industrial, small-scale).
- Fishery gear type (e.g. direct take, gillnet, longline, purse seine, squid jig/troll, trawl).
- Fishery target species (e.g. tuna, billfish).
- Regions of the Pacific Ocean (North, East, South, West, Central).

Further detail on the selection of reference articles can be found in *Additional information*.

The abstracts of all reference articles were put through word cloud generation software (https://www.wordclouds.com) to identify the terms most commonly used within these articles (e.g., 12, 15). This combination identified key terms from all articles and additional terms, based on the desired characteristics of bycatch data (SPIDER methodology) and the expertise of the authors were added to the search terms.

The Boolean search string was manipulated until all ten reference articles were retrieved from the database using only the search string. Ten random articles were chosen independently by searching “Pacific” “leatherback” “bycatch” in Google Scholar and selecting the first ten articles. These independent articles were used to ensure that our specific search string would also return more general articles, i.e. outside of the ones we hand-selected as relevant to our study, but relevant to the broad scope of this research area. The Boolean search string was validated after it also retrieved these ten independent articles.

### Creation of a boolean search strategy

Bibliographic databases SCOPUS, Web of Science Core Collection, and AFSA (Aquatic Science and Fisheries Abstracts) were searched with the following Boolean search.

(((marine AND turtle) OR (leather$back*) OR (leatherback) OR (critically AND endangered AND migratory)) AND (pacific OR (global AND overview) OR (east* AND pacific) OR (west* AND pacific) OR (ep AND leatherback*) OR (wp AND leatherback*)) AND (bycatch OR (fisheries AND interaction) OR (turtle-fisheries) OR (risk AND management AND tool) OR (rapid AND assessments) OR (direct AND take) OR purse-seine OR longline OR gillnet OR (small-scale AND fishery AND bycatch)) OR (pelagic AND trawler AND Atlantic))

This resulted in recovering 8/10 reference articles and 10/10 independent articles. However, there were two reference articles that were in grey literature and not retrievable in these databases when searching by their titles. This prompted the use of grey literature search in Google Scholar as described below, with smaller Boolean search strings targeting specific e.g. countries, then assessing the first ten resulting articles for inclusion. This methodology was able to retrieve the remaining articles. The combination of academic and grey literature search strategies yielded 10/10 reference articles and 10/10 independent articles. Therefore, this method was able to achieve our desired scope and will be suitable for this review.

## Types of literature

*Academic literature* will be screened via three relevant databases of peer-reviewed articles in scholarly journals, reports, books, and trade journals: SCOPUS, Web of Science Core Collection, and Aquatic and Fisheries Science Abstracts (AFSA). The Boolean search string will be adapted for that particular search engine.

*Grey literature* will be searched via Google Scholar. Instead of one Boolean search string, and in order to find articles specific to a fishery size, gear type, target species, or nation, several smaller Boolean search strings will be used. As a preparation for grey literature search, we compiled a list of key “factors” relating to leatherback bycatch for nations with fishing presence in the Pacific Ocean. The list includes factors for fishery size, gear type, target species, fishing nation, and the official language spoken by the fishing nation, in order to potentially locate information that may not be published in English.

- *Fishery size*: industrial, small-scale.
- *Gear type*: direct take, gillnet, longline, purse seine, squid jig/troll, trawl.
- Target species*: billfish, tuna.
  *Many target species are not listed here, particularly within small-scale fisheries.
  The Boolean search string does not require specific target species, but rather the term “bycatch”. All resulting target species will be recorded as metadata.
- Nation*: American Samoa, Australia, *Cambodia*, *Canada*, Chile, China, Colombia, Cook Islands, Costa Rica, *Dominica*, Easter Island, *Ecuador*, El Salvador, Fiji, French Polynesia, Galapagos Islands, Guam, Guatemala, India, Indonesia, Japan, Kiribati, *Malaysia*, *Marshall Islands*, Mexico, Micronesia, *Nauru*, New Caledonia, New Zealand, Nicaragua, Niue, Norfolk Island, *North Kore*a, Northern Mariana Islands, Norway, Palau, Panama, Papua New Guinea, Peru, Philippines, Pitcairn Island, Russia, *Samoa*,* Singapore*, Solomon Islands, *South Korea*, *Spain*,* Taiwan*, *Thailand*, Timor Leste, Tokelau, Tonga, Tuvalu, *Ukraine*,* United Kingdom*,* Uruguay*, United States, US Minor Outlying Islands (Midway Atoll, Wake Island, Johnston Atoll, Kingman Reef, Palmyra Atoll, Howland Island, Baker Island, Jarvis Island), Vanuatu, *Venezuela*, Vietnam, Wallis and Fatuna, *Yemen*.
  *Normal text denotes nations with Pacific Exclusive Economic Zones (EEZs); *italics* denote nations which do not have Pacific EEZs but do have fishing vessels in the Pacific Ocean according toThe Sea Around Us [29].
- Language*: Bahasa Indonesian, Bislama, Carolinian, Chamorro, English, Fiji Hindi, Fijian, Filipino, French, Hindi, Hiri Motu, Japanese, Kiribati, Mandarin, Māori, Marshallese, Niue, Norfuk, Norwegian, Palauan, Portuguese, Pukapukan, Rapa Nui, Rarotongan, Russia, Samoan, Spanish, Tetun, Tok Pisin, Tokelauan, Tongan, Tuvaluan, Vietnamese.
  *Based on a Google search of the official language of the fishing nations,.

Google Scholar search will be directed by mandatory keywords “Pacific”, “leather$back”, and “by$catch”, plus the addition of one of the keywords listed above, e.g. “American Samoa”. In the case of different languages, the keywords “Pacific”, “leatherback”, and “bycatch” will be translated into each language specified above and entered into a Google Scholar search. Articles will be sorted by relevance, and the top ten will be selected for translation. Translations will be performed using the DeepL translator (https://www.deepl.com/en/translator; [9]) from the language of the article into English. If the abstract, keywords, and title of these articles meet the eligibility criteria for inclusion, they will be added to the article pool for further assessment, as described in *Article selection based on eligibility criteria*.

Additional literature will be sourced through further methods (below), and the type of publication from which each estimate has come from will be reported.

1. Snowball sampling: identifying relevant articles and reviewing their cited literature, as well as contacting lead authors and inquiring about any additional key literature.
  - This could also be done in the form of contacting researchers through mail lists such as the CTURTLE listserv, and coordinators of research networks such as the Interamerican Tropical Tuna Commissions (IATTC), Bycatch Working Group, the Interamerican Sea Turtle Convention (IAC) Scientific Committee, the LáudOPO Network, and MigraMar.
2. Searching websites of fisheries management organisations and regulatory agencies: The names of multilateral regulatory agencies and regional fisheries management organisations (RFMOs) will be identified during literature search and their websites will be searched directly for relevant reports.
  - (e.g. the IATTC, Food and Agricultural Organization (FAO), Western and Central Pacific Fisheries Management Commission, South Pacific Commission, South Pacific Regional Environment Program)
3. Reviewing federal bycatch reports: Reports from fishing nations will be searched for in the official language of their nation and reviewed where available.

### Article selection based on eligibility criteria

Items sourced using the methods described above will be first reviewed by title, keywords and abstract, as provided when exporting references from database to EndNote. Each article will be screened against the SPIDER search strategy tool (Additional file 1, “Example.Datasheet”). Articles must satisfy the Sample, and Phenomenon of Interest, and Evaluation criteria upon review of the title, abstract, and keywords to be included in full text screening. In full text screening, articles must satisfy the data requirements for each Sample, Phenomenon of Interest, Design, Evaluation, and Research type, as listed below, to be deemed valid for inclusion in this study. Studies that satisfy all the criteria for validity will be included within these analyses to quantify the mortality of leatherback turtles in Pacific fisheries. These articles will be further analysed through critical appraisal, undertaken by the lead author using the CEE critical appraisal tool (Additional file 4; 21). If the appraiser is unsure about any article, an additional team member will assess the article.

### Data extraction strategy

Data extraction will occur after an article is deemed valid for inclusion and will include recording data on each of the following characteristics based on the SPIDER search strategy:

#### Sample:

Data from wild leatherback sea turtles will be prioritised where available. In the event that no leatherback-specific data exist, marine turtles will be sorted by species.

**Common name**, **Latin name**, **region of the Pacific**, lifestage, sex Phenomenon of interest conditions of bycatch.

**Gear type**, **interaction type** (if available: mouth-hooked, foul-hooked (external) or entangled), **effort involved** (number of vessels, length of time, area covered)

#### Design:

**where did these data come from**, and how certain is that data source? If there are multiple sources for one fishery estimate of leatherback bycatch, sources will be assessed to see if their published findings contradict each other or are synonymous.

**Certainty** will be noted using the scoring method from fisheries catch reconstruction [30] e.g. case study, experiment, interview, logbook, observer, report (govt), report (fishery), survey.

#### Evaluation:

quantified number of turtles **number interacted with**, fate of the turtles, number of morbidities and mortalities.

#### Research type:

quantitative; however, the datasheet will allow a space for additional and/or qualitative notes to be taken.

**Bold** above denotes data that are required to deem any article “valid” for inclusion within the meta-analysis upon full text review. All articles will have an individual record of why they were excluded from this analysis, showing their inability to satisfy one of the mandatory SPIDER search criteria. Further, data certainty will be scored to rate quality of each data source using fisheries reconstruction methods (30. In this method, a bycatch estimate built from multiple, published values would obtain a certainty score of 4, whereas less certainty would be attributed to the estimate if taken from a single published value “3”, an extrapolation, “2”, or if it was unknown and therefore an average from that gear type was applied “1”. In the event that published data are unclear, corresponding authors will be contacted for clarification. If clarification is unable to be provided, that article will be excluded from the analysis.

#### Bycatch estimation

To amalgamate the collected data into an overall estimate of Pacific bycatch, the number of turtles caught in each characteristic of interest (fishery size, gear type, target species, fishing nation) will be quantified. If no specific data are available in a particular combination, data may be extrapolated, although uncertainty will increase. For example, to obtain an estimate of leatherback bycatch for a Portuguese longline fleet in the South Pacific, it would be most relevant to extrapolate from a fishery with the same gear type/target species, as leatherback diving behaviour indicates that some gear types and target species combinations are riskier than others for leatherbacks [2]. Data variance (standard deviation, standard error) will be reported.

More specifically, to quantify bycatch and mortality for each fishery, the number of leatherbacks caught will be converted to a rate for each record, using metadata collected on the amount of fishing effort observed (i.e. number of hooks, nets, soak time, etc.). This first rate will be bycatch (and mortality) per unit effort (BPUE and MPUE): the number of leatherback turtles per 1000 hooks, 1 net, 1 seine, or 1 trawl. These rates will be used to compare risks to leatherbacks, and to identify fishing nations or gear types with significantly higher bycatch and mortality rates. Comparisons will be made with contingency tables and significance will be determined through chi-squared tests, or ANOVA (analysis of variance) where appropriate [13]. Additionally, total mortality per unit effort (TMPUE) will be calculated for each fishery as a combination of the literature mortality value, plus the bycatch value (number of turtles released alive) and the estimate of post-release mortality specific to that gear type [14]. While this does assume that post-release mortality values from the IATTC apply to the entire Pacific, it allows accounting for the leatherbacks that are released alive from fisheries interactions that suffer mortality later. As such, it is an important subset of data relevant for predicting the population trajectories using PVA.

Additionally, this study aims to scale up total mortality rates to an annual mortality estimate for leatherbacks in the Pacific fisheries. To do this requires an understanding of annual fishing effort in each fishery, which is difficult to estimate. Global Fishing Watch datasets are publicly available (https://globalfishingwatch.org) and record the daily activity of fishing vessels based on automatic identification systems. These data include the nation fishing, the gear type, and the number of hours spent fishing. While we recognise that these datasets are unable to capture all Pacific fishing vessels, especially small vessels, the data provide an opportunity to estimate total mortality with a novel method that more accurately reflects the amount of annual fishing effort and its impact on leatherback turtles. Should more data become available, the analyses should be redone. Using Global Fishing Watch datasets, and our metadata on the number of hours fishing, total mortality rates will be adjusted to “total mortality per fishing hour”, TMPFH. These rates will then be multiplied by the total fishing hours for that fishery in the Pacific, in the most recent year in which Global Fishing Watch data are available.

All proposed methods for literature review and bycatch estimation will additionally be evaluated through interviews with experts who have published in the research area of leatherback bycatch (estimation, modelling risk, or implications to fisheries management) to acquire their feedback. Experts will have an opportunity to provide feedback via a virtual interview in which these methods will be discussed, and a survey which will assign certainty scores to each component of our methodology. This follows certainty estimation methods from hydrogeological models [41] and will help generate the confidence intervals for the resulting PVA model updates.

### Potential sources of bias

The scale of fisheries bycatch data is broad, covering multiple nations, languages, regions, and gear types. Every article will be subject to screening by the lead author to determine its inclusion. Articles will be searched for in multiple languages, but translation discrepancies may create an unintentional bias. While academic and grey literature searches will be utilised in this work, there is potential that some literature will be unintentionally missed. The purpose of the additional “snowball sampling” is to identify when a saturation point has been reached (i.e. when our requests for additional literature and answered only with articles which we have already screen for validity within this study). Due to their expertise in the field of marine science and sea turtle conservation, the screener/s may have read some articles in their entirety. In these cases, care will be taken to review only the abstract and title for inclusion in the literature review. It is expected that any bias will be identified and minimised before publication of these findings. Potential bias will be further minimized by applying CEE Critical Appraisal Tool mentioned above (*Article selection based on eligibility criteria*; 21). This tool has been specifically designed by the Collaboration for Environmental Evidence to improve the rigor of synthesising scientific evidence by ensuring that decision making is based on high-quality evidence, and the risk of bias is thoroughly addressed within systematic reviews [21].

### Dissemination of findings

The results from our systematic review will be compared through a meta-analysis. All bycatch values retrieved from the literature review will be tested for normality, homogeneity of variance, and independence. Data which meet these requirements will be analysed through contingency tables, chi-squared tests, and analysis of variance (ANOVA) to compare the mean bycatch value by fishery gear type, fishing nation, fishery size, fishery region, and fishery target species. Additional insights will be reached by comparing the findings based on the data source.

The input and results from this work will inform a subsequent PVA, and both will be presented at a workshop facilitated by the IUCN Conservation Planning Specialist group in collaboration with Upwell Turtles (www.upwell.org) to inform management of Pacific leatherback populations. The results from the systematic literature review, the PVA, and the workshop report will each be published and available online.

## Electronic supplementary material

Below is the link to the electronic supplementary material.

Supplementary Material 1: Additional file 1 Scoping and independent article information including: List of abbreviations for criteria of interest for scoping articles; List of 10 scoping articles and how they satisfy the criteria of interest; List of keywords taken from scoping articles; List of independent articles generated to test search methodology.

Supplementary Material 2: Additional file 2 Example datasheet determine studies valid for inclusion.

Supplementary Material 3: Additional file 3 ROSES checklist.

## Data Availability

The datasets generated and/or analysed during the current study will be made available in the Dryad digital repository.
